# Digitalization of medical and healthcare education in a Low- and middle-income country: a mixed-method case study of institutional transformation at Yerevan State Medical University

**DOI:** 10.3389/fdgth.2026.1901577

**Published:** 2026-08-20

**Authors:** Karine Sargsyan, Tamara Sarkisian, Hasmik Tumanyan, Sergey Sargsyan, Khachatur Margaryan, Konstantin Yenkoyan, Larisa Avetisyan, Garnik Avetisyan, Armen Muradyan

**Affiliations:** 1 IBD Institute and OncoBiobank, Cedars-Sinai Medical Center, Los Angeles, CA, United States; 2 Vice Rectorate for Research, International Biobanking and Education, Medical University of Graz, Graz, Austria; 3 Department of Digital Medicine and Artificial Intelligence and Department of Medical Genetics, Yerevan State Medical University, Yerevan, Armenia; 4 Center of Medical Genetics and Primary Health Care, Yerevan, Armenia; 5 Students Association, Faculty of Medicine, Yerevan State Medical University, Yerevan, Armenia; 6 Yerevan State Medical University, Arabkir Medical Centre—Institute of Child and Adolescent Health, Yerevan, Armenia; 7 Vice-Rectorate for Development and Educational Group of Professional Competences and Healthcare Management, Yerevan State Medical University, Yerevan, Armenia; 8 Vice-Rectorate for Science and Neuroscience Laboratory—COBRAIN Scientific-Educational Center for Fundamental Brain Research, Yerevan State Medical University After M. Heratsi, Yerevan, Armenia; 9 Vice-Rectorate for Academic Affairs, Yerevan State Medical University, Yerevan, Armenia; 10 Vice-Rectorate for Post-Graduate and Continuing Education, Yerevan State Medical University, Yerevan, Armenia; 11 Rectorate and Department of Urology, Yerevan State Medical University, Yerevan, Armenia

**Keywords:** digital education, digitalization of higher education, digitalization of medical education, healthcare education, medical education

## Abstract

**Introduction:**

Digitalizing healthcare professionals' education for the future is essential to ensure the availability of skilled medical professionals across settings, particularly in low- and middle-income countries (LMICs), including underprivileged and disaster-hit regions. The process of translating theories into practice through digitization is challenging, and in this paper, we present a pathway based on Yerevan State Medical University (YSMU)'s experience with this process.

**Methods:**

The university has utilized constructive and activity-based approaches to integrate online learning, simulation, telemedicine, tele-education, and hybrid learning into its educational process. The preparatory work for the modern healthcare education approaches introduced in 2019-2022 has enabled the university to facilitate the transition to digital media, improve educators' and students' digital literacy, and develop evaluation methods that may help continue education during regional tensions and even throughout the COVID-19 pandemic, but also beyond, to intensify the learning experience with modern technology.

**Results:**

Student evaluation outcomes revealed a high degree of perceived satisfaction (overall satisfaction rate 90.2%) and self-reported improvement in theoretical knowledge, clinical skills, self-confidence, and digital literacy. The findings described student perceptions and institutional experiences of the respondents and not objectively measured improvement in learning outcomes.

**Discussion:**

This case study presents a single institutional route to overcoming infrastructure, technological, pedagogical, language, and equity barriers in digitizing medical education; whether this route can produce similar outcomes in other LMIC contexts remains to be tested.

## 1 Introduction

The digital revolution and the rapid—almost explosive—technological development in medical education encompass numerous aspects crucial to the ongoing evolution of teaching methods for future medical professionals. Digitalization changes the approach to medical education by transforming theoretical knowledge into practical skills through integrated systems, e-learning, and data-driven curricula (1).

Such factors include numerous theoretical models, which together determine appropriate teaching methodologies, as well as technological breakthroughs that have improved the learning experience and facilitated teamwork among both teachers and students. Efficient administration is important for the optimal realization of all teaching and learning activities. In contrast, certain pedagogical models can be used to engage students actively in meaningful ways, enhancing understanding of the subject matter (2). Moreover, this overall process would entail implementing major education reforms to improve curriculum development and its appropriate application. It is important to undertake such transformations in light of the perpetual changes in medical education (3). One of these major transformations should involve creating effective digital health systems that provide students with easy and fair access to relevant education and training resources and information to facilitate learning (4).

In addition to all the above, it is necessary to ensure effective learning outcomes for students and to provide a proper, generation–conforming education environment for future healthcare professionals. By implementing these transformations, one can be assured that students will not only acquire the relevant knowledge but also be equipped with the technical skills to succeed in their careers as healthcare providers in a technologically rapid developing, and technologically driven environment (5, 6).

### 1.1 Conceptual framework of digitalization in medical education

Digitalization is facilitated by rapid, almost explosive technological progress, which promotes educational processes and requires increasing focus on student-oriented digital teaching and on lifelong learning needs (1, 7).

Pedagogy has undergone significant changes over the past few decades, shifting the concept of education from a traditional information-dissemination model from one (teacher or lecturer) to many (big groups of students) to personalized, student-centered approaches in which learners actively construct their own understanding of subjects (8).

Constructivism in the medical context involves meaningful, authentic learning activities set in real-world contexts (1). Education should therefore engage learners in real-world tasks rather than present artificially restricted tasks divorced from the field. Learning should also be situated within a context relevant to the field being studied. In this regard, the concepts of problem-based learning (PBL) and scenario-based learning (SBL) were introduced into situated learning, leading to the derivative concept of activity-based learning (ABL), which describes the cycle of learning through action and reflection on situated practices (9). As digital communication devices became mainstream, many transaction-based and activity-based pedagogies were introduced, enabling students to construct knowledge actively through partnering, externalizing dialogues and reflections, and connecting and sharing digital content across different spaces, contributing to the rise of pedagogy 2.0 on a global scale. The pedagogy 2.0 paradigm extends beyond educational institutions, promoting the beginning of lifelong learning involving multiple participants and organizations (6).

### 1.2 Historical overview and rationale

Investment in medical education is being made continuously and sustainably in Armenia, specifically at YSMU. On one hand, there is a strong medical education knowledge with heavy reliance on traditional teaching methods, especially in clinical settings; on the other hand, there is a need for a transition from teaching to learning, without restricting the development of newly acquired technologies and with them coming knowledge and skills in line with internationally recognized 21st-century standards (6).

Explanations of educational difficulties focus either on sociological problems or on the lack of up-to-date teaching materials. Although sociological factors—such as those that contributed to the widespread adoption of the Internet in developed states—are perceived as obstacles to the introduction of modern educational technology in Armenia, approaches that have worked in similar countries could still facilitate implementation (10–12).

The first steps in bringing medical education into the digital era have been taken since early 90s. Nevertheless, societal turmoil continues to curb the infusion of information technology into teaching programs. In an attempt to modernize Armenia's educational system in this area, constant monitoring reveals that excessive reliance on traditional information flow processes persists.

### 1.3 Theoretical models and pedagogical approaches

A broad, heterogeneous array of theoretical frameworks has been proposed to describe and conceptualize digitalization in medical education. Additional consideration of pedagogically equipped virtual learning environments is an efficient means of enhancing access to human resources in developing countries, where the availability of qualified medical fit facilitators is often restricted. The need for remote medical education amid socio-political disturbances further underscores the importance of addressing this topic (6). The pedagogical models of social constructivism, connectivism, and experiential learning are equally relevant to considering the convergence of digitalization, higher education, and the associated proliferation of pandemic-related socio-political studies (13). A paradigm shift in the field of digital medical education is anticipated, as cloud-based and open-source platforms such as the metaverse and XTZ enable the immersive representation of real-life, complex scenarios and virtual human interactions, unrestrained by space, time, or matter (7).

## 2 Digitalization in the Armenian context: YSMU

The digitalization of medical and healthcare education covers lots of ground, too. It examines learning theories and teaching methods and addresses the design and analysis of learning spaces. Also, there's modern curriculum planning, assessment strategies, and faculty training. We mustn't forget to manage students and measure learning success either. The shift process description from a highly theoretical to a more practical method of analysis can be done by considering a specific institution- particularly YSMU, which represents the main medical university with over a hundred years of long history of educating the healthcare professionals (6). YSMU's strategic priority is to digitize the delivery and management of medical and healthcare education—specifically in pre-med, but also in other knowledge-driven aspects, while keeping a hand on the excellence of clinical education. The specific institutional strategy was outlined and implemented alongside the supporting infrastructure to underpin it (14–17). The transition is organized according to the cyclical framework of educational design, delivery, and management. The framework comprises the design of the curriculum and educational program; the digital-competency enhancement and general professional development support offered to faculty; the concurrent development of student digital literacy and engagement; and, ultimately, the organization of pedagogically rich, digitally enhanced learning opportunities (12, 15).

### 2.1 Institutional strategy and policy in short

At YSMU, issues related to digital health literacy are currently being addressed within several existing modules for medical students. These modules include structured discussions on the practical and ethical use of digital health tools, such as telemedicine platforms, electronic health records, and mobile health applications, as well as fundamental aspects of health data management and cybersecurity (18). The topics have been analyzed using case studies, problem-based learning, and certain practical demonstrations. Internal observations suggest that students are increasingly aware of the application of digital technology across both clinical and public health fields. This highlights YSMU's effort to introduce digital health into undergraduate medical education and provides a basis for academic review and dissemination of findings (14, 19).

### 2.2 Plan: infrastructure and technological ecosystem

With the rapid, widespread introduction of complex digital technologies into societies, educational processes have also begun to change. Digitalization has undeniably influenced medical and health education and continues to be integrated into medical universities and educational institutions worldwide (6). Digital technology also offers developing societies the potential to create interactive networks. In Armenia, digitalization can support the establishment of links with the global medical and health education system, further strengthened by significant post-Soviet developments in curriculum reform. The aim is to foster a health education system that adopts an evidence-based approach to quality design. Digitalization of health education requires critical reflection on both theory and practice, as well as proactive analysis of how to implement these initiatives to benefit Armenia (20). Digitalization involves diverse infrastructures and networks, including, but not limited to, the YSMU information system's computer hardware and Internet connectivity.

An important initial step for medical education is to prepare the learning environment and adapt the information system to local needs and specific infrastructure challenges. Health education that addresses the identified framework will support future medical education curricula. Critical transparency among national and global stakeholders, along with ongoing revision and improvement, contributes to the realistic, widespread, and sustained introduction of health education. Failure to achieve these objectives might compromise the proper and maximally ethical implementation of these potentials (18–20).

### 2.3 Combining digital media in curriculum design

Digital competencies are being rapidly integrated into the existing curriculum; this is a strategic priority for YSMU and is reflected in the current staff development policy. Three essential competencies in this context are Digital Learning (the skill to use digital technologies for learning), Information Literacy (the skill to evaluate the nature and quality of information), and Communication and Collaboration (the skill to engage with others, including the capability and skills of communicating effectively in an online context). The objective is to facilitate staff and student engagement on campus and increase the ability to adapt existing courses to hybrid and blended formats (6). Increasing the availability of online resources, even in fully on-site learning programs, is essential to fostering digital literacy and expanding access to digital transformation training. The expected post-COVID return-to-campus period promoted the development of innovative approaches to learner engagement (19, 20).

### 2.4 Faculty development and digital competencies

Digitalization and modernization of the medical and healthcare education sector are among YSMU's main priorities. There has never been a greater need for academic system reform, considering not only educational quality but also the effective allocation of educational time and space (6). Digitalization offered a relevant solution to the problems in the education system and to the tremendous challenges posed by the COVID-19 pandemic. Efforts in this field continue to accelerate: YSMU has embarked on a two-year journey—January 2021 to December 2022—aimed at accelerating the transition from the theory of digitalization in medical education to practical implementation (10, 18, 20). In parallel, developments in telemedicine, tele-education, and remote supervision are also being incorporated into educational programs. Digitalization comprises continuous, innovative development of content & curriculum, as well as pedagogical approaches. It is critical, however, to have already existing strategic planning in place. As a priority, faculty development and the digital competencies of all stakeholders have been established as prime activities. YSMU has prepared competent staff in this respect from the outset.

### 2.5 Plan: student engagement and digital literacy

The process of digitalization in education will be accelerated by increased student engagement and improved digital literacy among medical and healthcare students (6). The Armenian context is unique: a protracted Soviet legacy has stalled reforms in the curriculum, pedagogy, and assessment methods that have long been essential to the modernization of medical education. Globally, among the factors most frequently cited by students as impeding their engagement in and readiness for digitalization are a lack of internet access, inadequate relevant knowledge and skills, a lack of digital competence, and a lack of time (21). A recent assessment of the level of digital literacy necessary for engagement in and readiness for modern medical education in other settings is therefore very timely.

### 2.6 From theory to practice: case studies at YSMU

Medical education is a highly relevant and significant factor influencing the development of the national healthcare system. The need and the calculated number of doctors and medical personnel are indicated not only per capita but also by the incidence of common diseases in the population. As a developing country, Armenia faces challenges in its health system. The quality of medical and healthcare education significantly affects Armenia's health and well-being (6). Yet digitalization was not prioritized in the Armenian medical and healthcare education system in the beginning of 20th century —including higher, postgraduate, and continuing education (20).

#### 2.6.1 Virtual learning environments and e-learning platforms

E-learning and online teaching tools let med students, teachers, and health pros talk, swap ideas, chat about cases, and team up on research around the globe. The creation of a strategic implementation plan would enable organizations to use new technologies effectively, address problems as they arise, and support effective learning. The use of technological innovations has been an important part of medical education for some time now, with current advancements including simulations, virtual patients, and computer-based patient records, which have improved learning and healthcare (7). The global coronavirus pandemic forced educational institutions worldwide to move from traditional classroom learning to online learning, resulting in adverse effects on learners' well-being.

Educational institutions revised curricula to suit the new conditions, requiring all interactions between teachers and students to take place via technological means and mandating that senior students receive additional training in hospitals. Curriculum revisions that included active learning strategies facilitated the transition to online classes through technologies such as video conferencing platforms and e-learning management systems. Russia is apprehensive about distance learning, particularly regarding the acquisition of clinical skills. It is crucial to receive feedback from students regarding their remote learning experience (22).

#### 2.6.2 Simulation-based education and clinical skills

The advantages of simulation-based education include gaining real-life experience and developing techniques through practice on a phantom, mannequin, or computer-based simulations to implement protocols for providing care in urgent cases. The training helps health-care professionals practice techniques not on patients but in realistic situations. They learn practical and communication skills within a team, which helps improve quality. Since 2018, such programs have been introduced at YSMU in three medical faculties. The education program includes specialized simulation, basic and advanced skills, and case-based learning. New distance classrooms and telemedicine classrooms with conferencing systems are used for remote supervision of students at YSMU. The digital connection between YSMU and the other six institutions allows for dual degrees, collaborative learning, and research.

#### 2.6.3 Telemedicine, tele-education, and remote supervision

Tele-education, remote supervision, and telemedicine programs have become priority areas as a digitally enhanced educational process is introduced. They help continue teaching and supervision across many subjects when face-to-face meetings are impossible due to various conditions, such as the coronavirus pandemic that began in 2019 or a war in Artsakh. In addition, due to the emergence of a digital educational platform, tele-educational services and supervisory systems have been established to enable students to benefit from experts' experience.

The following are some tele-education forms of YSMU programs: on-site education with intervals for urban students, delivered via distance instructors at the university and other educational institutions; courses in which students attend online classes organized specifically for students at YSMU's regional branches; and free online courses provided for healthcare professionals and community members. Using both models of tele-education, specifically through direct supervision and consultations, will make the entire procedure more efficient. Specifically, the tele-consultation model was used to connect students with clinical cases related to coronavirus infections.

#### 2.6.4 Assessment in a digital era

The adoption of new digital assessment systems can lead to changes in examination modes. The educational activities and examinations during the COVID-19 pandemic were modified to be administered via Zoom, in small study groups, and through online tests. Alternatively, new assessment forms that better align with the digital paradigm—such as flipped classrooms, open-book testing, and individual or group work submissions—were considered and designed. To complement such formative evaluations, YSMU continues to conduct summative assessments at the term's end. Since 2018, Yerevan State Medical University (YSMU) has benefited significantly from digitalization efforts in the fight against the challenges that appeared during the coronavirus pandemic. It has established a comprehensive digital environment for educational telemedicine applications (6, 13). However, introducing digital exams involves some risks.

The immaterial nature of digital examinations makes it more difficult to distribute summative examinations. Easy access and distribution can promote fraud. Detecting cheaters can be difficult due to Zoom's limited feedback capabilities (e.g., quiz or real-time question-and-answer tools). Various forms of summative assessment are being developed to address these problems in line with national, institutional, and departmental policies. Faculty and administrative staff committees are currently responsible for these efforts. As a result, an educational institution can be flexible in responding to epidemiological conditions and further develop its pedagogy and testing practices. In Armenia, all legitimate evaluation methods require state accreditation to ensure effective knowledge transfer. The Yerevan State Medical University uses digital methods that pass this test as well. Per Armenia's Ministry of Education, even as it uses new tech, core learning can't be ignored or treated as separate from regular classes. There's regular checking to ensure the group's work aligns with the curriculum.

## 3 Methods

**Figure 1:**
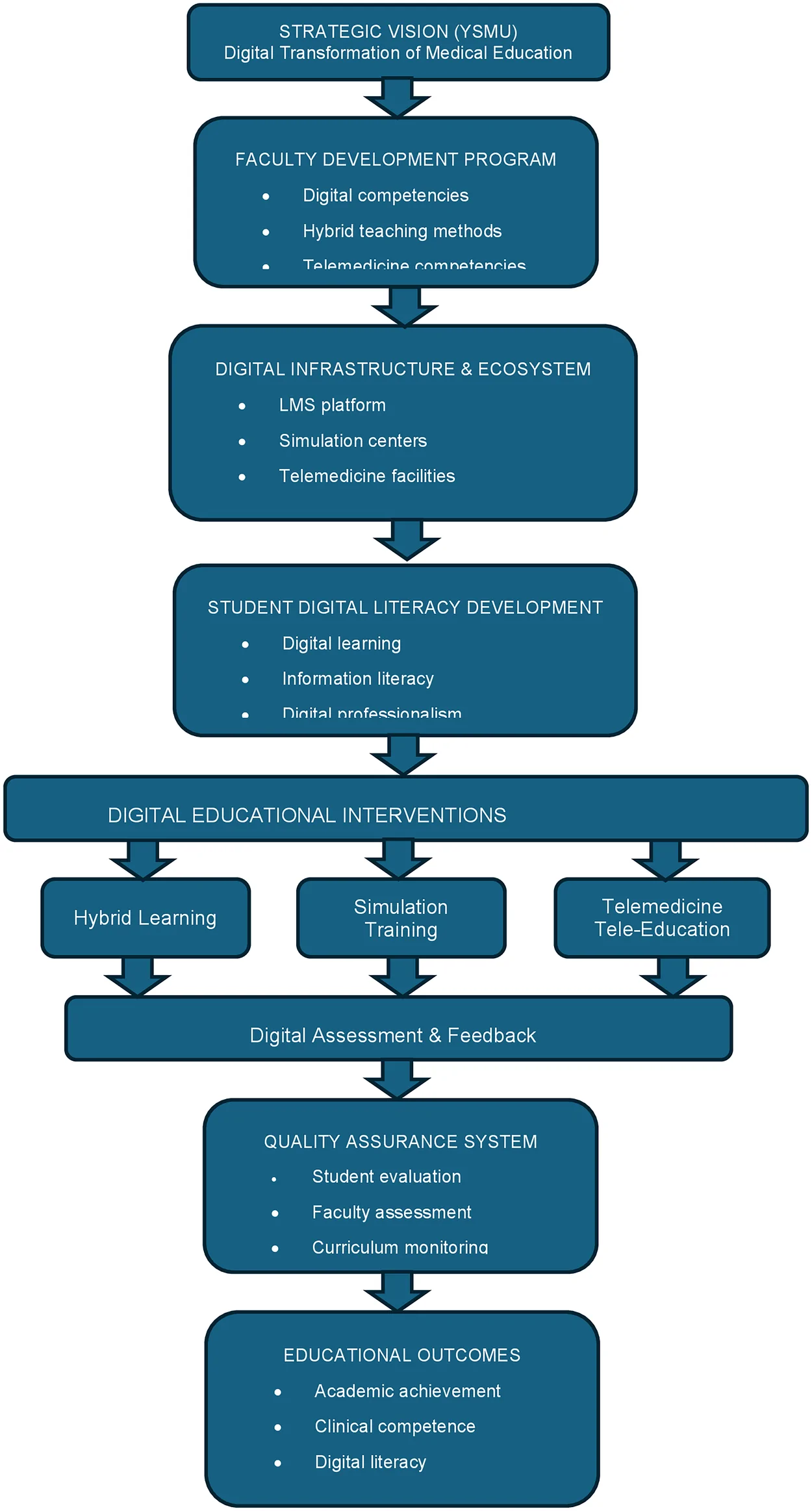
YSMU digital transformation framework (2017–2025): From theory to practice in medical education, From strategy to educational outcomes.

The primary approach is institutional development and implementation of the digital education framework. The student survey provides useful, supportive information: (a) the survey serves as an evaluation component of the transformation process, and (b) the central contribution remains the institutional experience itself. The digitalization of the educational workflow at YSMU has been carried out in accordance with Figure 1.

*Study Design*: The study was designed as a mixed-methods institutional case study in the context of the COVID-19 pandemic to maintain high teaching standards and to evaluate the implementation and outcomes of digitalization initiatives at Yerevan State Medical University (YSMU) between 2019 and 2023. It took part in parallel with the university's standard education evaluation efforts as a higher education institution.

*Participants*: All undergraduate medical students enrolled in years 2–6 at YSMU were asked to complete the questionnaire (total: 4,080). A total of 513 students voluntarily completed the questionnaire (12.6%—which is quite respectable for a voluntary evaluation questionnaire).

*Data Collection*: Data collection was performed in Fall 2023 via distributed questionnaire anonymously and electronically, The questionnaire comprised eleven Likert-scale items evaluating most significant aspects of digitalization in medical education, like satisfaction, digital literacy, simulation-based education, telemedicine, tele-education, hybrid learning, and digital assessments. Participants could additionally provide open comments.

*Statistical Analysis*: For this case study with mixed methods most effective was found descriptive statistics—rates were calculated for all survey items. Results are given as frequencies, percentages, and mean scores. Because the purpose of the study was exploratory and descriptive, no inferential statistical testing was performed. This questionnaire was designed by the institution's working group according to the digitalization framework, while the content of this questionnaire was approved by three educators of the institution with extensive experience in medicine and digital health. The internal reliability of the 11 questions of the Likert scale is quite high (Cronbach's *α* = 0.87). As the questionnaire was designed for exploratory evaluation of the institution and not for cross-institutional validation, the factor analysis is not required.

*Ethics*: Participation for all 2nd to 6th year undergraduates was voluntary and more importantly—anonymous. No person related and/or identifiable information was collected. The study evaluated educational processes and institutional quality improvement activities. All data/response collecting procedures complied with institutional ethical standards of YSMU and the principles of the Declaration of Helsinki.

### 3.1 Importance of quality assurance, evaluation, and outcomes

Quality assurance and evaluation processes, including the development of quality assurance systems and the use of evaluation methods with appropriate metrics, play an important role in estimating the level of digitalization achieved. It can be stated from the students' evaluation and institutional observation that there was an association between digitalization initiatives and high levels of perceived satisfaction as well as gains in digital literacy and confidence in clinical skills; however, those findings are based on perceptions rather than objectively measured learning outcomes, clinical skills, or patient care quality improvement (6, 14).

The Armenian healthcare system also faces challenges related to equity, accessibility, and quality of care in rural regions. In addition to the challenges faced by Armenia, the quality of education amid armed conflict, the coronavirus pandemic, and other emergencies, including the migration of skilled professionals, should be considered and addressed.

To mitigate these issues, continuity measures were developed, including pedagogical approaches that foster the development of higher-order skills at YSMU (23). Educational research shows that pedagogical practices focused on higher-order skills can develop lower-order skills. This practice helps ensure the quality of the pedagogical practices used to develop higher-order content skills. The above innovations and pedagogical practices underscore the need to implement a quality assurance mechanism for the education process, which is incorporated into the recently developed national digital development strategy. The rapid development of information and communication technology, artificial intelligence, and the increasing relevance of remote services are constantly changing environmental conditions; therefore, the main elements of decision-making need to be acknowledged and strengthened (24).

### 3.2 Quality assurance frameworks

The quality assurance framework is designed to ensure the quality and efficiency of curriculum development. The key feature of the evaluation methodology is that it determines whether the program material exists and how well it meets all required criteria. Furthermore, it is possible to define the types of information needed to determine the score for both the individual and the group, based on those criteria. In the field of medical and healthcare education digitalization, one must measure the transition from theory to practice, including institutional aspects, applied methods, and achieved outcomes. A case study was conducted on the process of digitizing course preparation at YSMU for undergraduate and Master's programs. Both before and after the course, surveys helped establish whether the necessity of such digitalization, related challenges, and its effects at YSMU have been felt. The gradual implementation of e-learning in YSMU during the pandemic sparked interest in possibilities (6). The questionnaire administered before the training determined the number of hours traditionally spent on exam preparation. At the same time, the evaluation of performance on clinical tasks online measured the influence of online course delivery without increasing learning time.

Quality assurance frameworks are available at the international, national, and regional levels through professional and disciplinary associations. At the institutional level, the United Nations Educational, Scientific, and Cultural Organization (UNESCO) suggests six dimensions of quality assurance—governance and management; financial support; systematic planning for educational and learning processes; teaching and learning environment; student support; and monitoring, assessment, and continuous improvement—divided into related criteria (6, 22, 23). The Quality Assurance Agency for Higher Education (QAA) in Great Britain offers a five-point framework consisting of subject, information, enhancement, credit, and degrees. The World Federation of Medical Education's valuation criteria include the following five areas: educational strategy, educational process, educational resources, student evaluation, and professional development. The criteria of the European Association for Quality Assertion in Higher Education were considered when integrating digitalization into healthcare education (25).

### 3.3 Education evaluation methods

The analysis of the digitalization process, from theory to practice, in the field of medical and healthcare education will entail considering various factors, including the institution's history, the methodology used, and the outcomes achieved. In this regard, the method used to prepare for online courses in medical education was evaluated at YSMU. The need for this particular shift towards digitalization, along with its challenges and the effects it had on YSMU, was discussed based on pre- and post-course surveys on knowledge and efficiency in clinical cases. With the gradual development of e-learning in medical institutions of Armenia amid the coronavirus pandemic, there was an interest in going beyond theory (6, 13). To evaluate the effect of online courses on clinical practice, a pre-training survey was conducted to determine the time devoted to exam preparation and to assess task evaluation via online guidance.

### 3.4 Impact of digitalization of medicine and education methods on learning, clinical competence, and patient care

The impact of digitalization on educational approaches on student learning outcomes is regularly assessed. Analyzing questionnaires distributed after classroom activities helps identify early indicators of the teaching methodology's effectiveness and provides feedback for further improvement. A compulsory “Educational-Social Analysis” is included in the Teacher Portfolio Assessment documentation at YSMU. This is a comprehensive questionnaire with statements about the quality of the educational process (10). Student responses to that questionnaire indicate that they perceive the effectiveness of the digitalization process positively (6, 12, 19). No more than 10% of respondents reported a negative impact of digitalization on students' academic achievement.

The third form of evidence used to demonstrate how digitalization influences the acquisition of student learning and the development of clinical skills is an observational study of second- to sixth-year students undergoing their first clinical skills training in Fall 2023. Various medical and healthcare curriculum areas have been selected to develop students' professional competencies throughout the educational process, and Faculty Activities encompass trauma, obstetrics, and biomedicine. Questionnaire results from all 513 students at YSMU show that overall satisfaction and outcomes were strongly aligned with high levels of perceived improvements in academic performance and clinical skills.

## 4 Results

The questionnaire presented below (Table 1) was distributed to YSMU students (2nd–6th years, Faculty of Medicine) for voluntary completion following the 2019–2022 digitalization initiatives. The aggregated results are shown in Table 1.

**Table 1:** Satisfaction of students about digitalization activities at YSM.

Question/statement	Strongly agree (5)	Agree (4)	Neutral (3)	Disagree (2)	Strongly disagree (1)	Mean score	% Positive (4 + 5)
1. Overall satisfaction with digital/hybrid learning	314 (61.2%)	160 (31.2%)	28 (5.5%)	8 (1.6%)	3 (0.6%)	4.51	92.4%
2. Digital tools improved theoretical knowledge	289 (56.3%)	175 (34.1%)	34 (6.6%)	11 (2.1%)	4 (0.8%)	4.43	90.4%
3. Simulation-based training improved clinical skills	301 (58.7%)	163 (31.8%)	31 (6.0%)	13 (2.5%)	5 (1.0%)	4.45	90.4%
4. Telemedicine/tele-education effective during disruptions	268 (52.2%)	185 (36.1%)	42 (8.2%)	12 (2.3%)	6 (1.2%)	4.36	88.3%
5. Digital literacy/confidence increased significantly	308 (60.0%)	158 (30.8%)	32 (6.2%)	10 (2.0%)	5 (1.0%)	4.47	90.8%
6. Hybrid learning is more flexible	325 (63.4%)	143 (27.9%)	30 (5.8%)	9 (1.8%)	6 (1.2%)	4.50	91.2%
7. Experienced significant technical barriers (reverse	26 (5.1%)	48 (9.4%)	59 (11.5%)	165 (32.2%)	215 (41.9%)	1.85	14.4%
8. Faculty were well-prepared for digital delivery	252 (49.1%)	190 (37.0%)	47 (9.2%)	18 (3.5%)	6 (1.2%)	4.30	86.2%
9. Digital assessments were fair and effective	241 (47.0%)	193 (37.6%)	54 (10.5%)	17 (3.3%)	8 (1.6%)	4.26	84.6%
10. Digitalization positively impacted academic performance	282 (55.0%)	172 (33.5%)	40 (7.8%)	13 (2.5%)	6 (1.2%)	4.39	88.5%
11. Would recommend YSMU's digital approach	304 (59.3%)	165 (32.2%)	31 (6.0%)	9 (1.8%)	4 (0.8%)	4.48	91.4

Mean responses varied between 4.26 and 4.51 with the highest one for the satisfaction with the digital/hybrid learning in general and for hybrid flexibility (both 4.51), while the lowest one for the technical barriers (1.85, reverse coded). The percentage of a positive response (Agree and Strongly Agree) was 90.2%. The highest mean value of responses for the simulation training was observed among students in their clinical years (year 4–6) with the mean score 4.57. These values show subjective responses to digitalization and should not be considered as an objective evaluation of students' academic performance.

Despite this, the most concerning area wasn't even about fairness or effectiveness; it was tech issues. These problems got the lowest score at 1.85, showing that students didn't see these troubles as a huge hurdle, though clearly, there is room for improvement in that area.

The overall Average Positive Response Rate, as shown in Table 1, was 90.2%. The breakdown of all students (*n* = 513) by gender was almost balanced: 59% female (303) and 41% Male (210). The study year was evenly distributed across the 6 years of medical education (2nd-year students: 18%, 3rd-year students: 24%, 4th-year students: 27%, 5th-year students: 19%, and 6th-year students: 12%) and showed no reliable correlation with evaluation results. The study topic, or faculty of responders, was primarily general medicine (88%), with smaller numbers from dentistry, pharmacy, and public health/leadership studies. Interestingly, the so-called clinical-year students (4th–6th years) rated simulation-based training the highest (mean score: 4.57). Also worth mentioning is that female students showed slightly higher satisfaction with hybrid flexibility (mean 4.53). Most of the students didn't use the comment box, but we received some comments (translated into English by the authors). Part of the comments were very positive, like the following:

- “The simulation center of our university is extremely helpful to make me feel more confident in clinical scenarios now.”
- “Hybrid lectures allowed me to review complex topics multiple times, while managing my schedule onsite.”
- “Telemedicine sessions with specialists during the pandemic were very useful.”

Nevertheless, we received also some constructive comments, like:

- “Occasional internet instability during peak hours in Yerevan was a major restriction.”
- “Some professors need more training on the digital platforms.”
- “Would like more materials available in Armenian and in English, in parallel.”

## 5 Challenges and mitigation strategies

The establishment and integration of digital/virtual learning opportunities into YSMU's curricula open the way for their development and expansion. Participants identified educational and organizational challenges, legal-ethical barriers, pedagogical limitations, and infrastructure-related issues as drawbacks of implementing virtual education in a medical university. Addressing these challenges could move a medical university toward successful virtual education. Infrastructure, pedagogical, and legal-ethical challenges have captured the attention of the ministry and the country's higher education science center, prompting them to determine whether to continue virtual education or halt it completely. Such barriers should therefore be taken seriously into consideration when developing efficient strategic models for medical universities (26, 27).

### 5.1 Challenges: technological and infrastructural

Medical education is moving from traditional, teacher-centered approaches to learner-centered systems with greater diversity in organizations and modes of education delivery. Change is accelerating through new communication methods, educational technologies, and educational innovations. Medical schools have begun adopting new methods for conducting their educational practices and exploring other ways to develop their curricula and define educational objectives. It should be noted that the medical education system in Armenia has long been characterized by traditions, which relied heavily on a specific model of competency-based learning and quality assurance. Digitalization of Medical and Healthcare Education offers opportunities to address these shortcomings and strengthen the quality of training in an evolving landscape (6, 13).

### 5.2 Change management

When moving from traditional to digital modes of teaching, change may face significant resistance from teachers. However, this does not apply only to YSMU. As noted above, the analysis identified several obstacles stemming from teachers' unwillingness to digitalize, both at the level of particular disciplines and at the level of the entire institution, as well as differing views on their role in this process. As it turned out, even though most people working at the institution, both older and younger, realize that digital technologies have significant potential to improve education, not everyone does. Digital literacy initiatives seek to address these problems through discipline-based support and an institution-first approach. It should be mentioned that, without institution-driven motivation, “there will be no motivation among the educators to participate.”

Additionally, the mere absence of digital resources can impede effective efforts to assist colleagues in the initial stages of digital change. Therefore, addressing fundamental issues at the systemic level is essential for clarifying pathways to the initial stages of institutional digital transformation (27, 28).

### 5.3 Equity, accessibility, and inclusivity

An ideal educational system is accessible to all, regardless of gender, ethnicity, age, religion, disability, or any other characteristics. In Armenia, educational access is a national priority, but the issue is complicated by factors such as digital illiteracy and the availability of literature and technological means. All socio-economic issues are accounted for in the digital transformation process, particularly within LMIC (27).

### 5.4 Data privacy, security, and ethics

The digitization of health care platforms enables the monitoring of the quality and distribution of educational content in unparalleled detail. So, if the benefits outweigh the risks of data privacy and ethical concerns, they must not be overlooked. The enactment of legislation governing an individual's digital life (e.g., the Digital Personal Data Protection Bill, 2023) reinforces ethical considerations regarding the collection, exchange, and use of even anonymized data.

There is a significant gap in the training of digital ethics, privacy, and security (28, 29). The intersection of ethical, legal, and privacy issues often leads to confusion. In general, the legal procedures for healthcare research are poorly understood, and the legal qualification of an ethical act is seldom taught in higher education institutions. Ethics committees’ guidance is typically sought only at the final stage of research preparation, reinforcing the notion that legal knowledge is unnecessary in the education of healthcare professionals.

## 6 Limitations

There are several limitations to discuss. Firstly, it should be noted that this study is a single-institutional one and, therefore, lacks external generalizability of results, while providing internal consistency of context and process description. Secondly, this evaluation is based mostly on voluntary perceptions reported by participants rather than on objective indicators of their knowledge, skills, and patient care outcomes. Third, no contemporaneous comparison (control) group was available. Fourth, the response rate was low (513/4,080 = 12.6%). Although the demographic profile of respondents (59% female, balanced distribution across study years 2–6, predominantly Faculty of Medicine) is presented, no data on non-respondents were available; therefore, the possibility of non-response bias cannot be excluded and may affect the representativeness of the satisfaction scores. Finally, due to the nature of the design, any possible perceptual changes observed during the process cannot be attributed to digitalization initiatives introduced into the institution.

## 7 Discussion

This mixed-methods case study provides an example of how a visionary strategy of the institution, together with faculty development and relatively low-level investments into the infrastructure, can facilitate hybrid learning, simulation training, and tele-education even at the middle-income institution that is dealing with resource issues and regional instabilities. According to students' survey results, there are high levels of perception satisfaction and improvement in digital and clinical skills literacy.

### 7.1 Policy implications and strategic recommendations

The role of policy at the national and institutional levels in translating theory into practice is immense. Wherever there is an approach that encourages innovations in pedagogy and curriculum design, the use of technology is quite straightforward (6, 13, 18). Conflicts arising from inconsistencies between the nation's developmental objectives and the institutions can adversely affect the implementation process. The digitization project at YSMU is highly consistent with the nation's objectives; however, certain elements, such as telemedicine, have not yet been recognized in legislation. It was prioritized to incorporate more recommendations on service delivery and socioeconomics into the university's strategic framework, even before 2019. Although YSMU has achieved commendable milestones in its digitization journey, there are still areas that need further development (18, 20).

The strategic roadmap was prioritized. The process of digitalization continues to advance across various areas as ICT advances. The phenomenon of digitalization has caused a paradigm shift in medicine and health care education. To facilitate change at YSMU, a strategic roadmap has been devised to balance the organization's mission, the digitalization process, and expected results through 2025 and beyond.

YSMU is Armenia's largest and oldest dedicated medical university. It has adopted an ambitious mission to educate highly skilled healthcare professionals who can provide safe and effective health care, grounded in scientific evidence and personal commitment. Medical education is defined as a systematic and coherent series of planned learning experiences. The educational establishment's objective must be achieved by improving the curriculum and learning process through technology-based measures. Indeed, the digital revolution has brought substantial changes to education and training, and new approaches to improving the efficiency of learning interactions have emerged, realized through proactive and interactive techniques.

The process at YSMU can be viewed as one possible route through which the institution may pursue digital transformation in its initial stages in conditions of middle-income country and geopolitical constraints. The transferability of this particular model to other institutions or LMICs is a matter for hypothesis generation rather than fact. Simultaneously, a holistic strategy for digitalization of medical education has been elaborated and adopted as an appropriate response to modern twenty-first-century challenges. Alongside, examples of digitalization in medical education and training are presented, indicating their alignment with general development and digitalization of higher education in the country, the strategic plan of YSMU, and the multidimensional framework, with varying degrees of freedom. This analysis establishes and clarifies extensive adherence to a comprehensive institutional roadmap for the digitalization of medical and healthcare education at YSMU, guiding transitional directions and plans until 2026 and beyond (6, 12).

### 7.2 Lessons learned

The digital transformation at YSMU really highlights how education can be innovatively improved. It isn't just about overcoming technical issues; it's more about organizational and cultural readiness for change. Although having the right technology and resources matters, the real kicker at YSMU was that everyone—from leaders to students—was on board and dedicated to the changes.

- Institutional digital strategy must be tightly aligned with the educational mission; technology alone is insufficient.
- Sustained faculty development and ongoing peer support are essential; without them, even well-resourced platforms remain under-utilized.
- Students were instrumental to successful implementation at YSMU; instead of just keeping students informed of digital changes implemented in the classrooms, it was highly effective to include student feedback throughout the entire process actively. This participation contributed to student satisfaction on one hand and to program success on the other hand.
- Learning through simulation-based education and using telemedicine has significant potential to provide students with practical opportunities to build skills and leverage resiliency in times of disruption. The benefits of using these modalities would not be limited to emergency situations; rather, there is likely to be long-term value from incorporating simulation-based education and telemedicine into healthcare education.
- Additionally, an observation from Yerevan State Medical University (YSMU) was regard to the digital transformation as being a “continuous transformation” with ongoing commitment from institutions towards developing their digital capacity, as there are continually new forms of technology being introduced as educational needs are changing regularly, ultimately institutions will need to adapt and improve based on regular evaluation of their approach to be able to sustain their digital transformation.

Also demonstrated from YSMU is the balance between innovation and inclusivity; efforts to enhance access to educational experiences, through providing multilingual learning resources and supporting students with different digital literacy levels, were critical to ensure that technological advancements did not widen gaps of inequality.

### 7.3 Implications for low- and middle-income countries

This is a case study of a single institution; however, certain contextual issues such as resource limitation, infrastructural limitation, resistance to change, and the issue of maintaining education during emergencies are common among other medical schools operating in LMICs. It is possible that the process findings described in the study could be transferred; however, the combination of interventions and the high level of self-satisfaction cannot be generalized until more institutions are examined.

Therefore, this work may primarily align with developing countries within the post-Soviet region, Eastern Europe, Central Asia, and the Caucasus, as healthcare education systems in these areas are typically very strong in terms of traditional higher education relevance but face demands to modernize their healthcare education systems to create better global connections. In addition to post-Soviet organizations, it would also make sense to look at the lessons that could be derived from the experience of organizations from Africa or other developing countries, especially in terms of technological resources. As it turned out, transformation could be successful despite the lack of the most advanced systems. Factors contributing to success include strategic planning, involvement of all stakeholders, professional development of teachers, and implementation of quality control mechanisms.

The other aspect related to the experiences of developing nations is the application of digitalization for enhancing educational resilience. Considering current crises—namely the coronavirus outbreak and regional wars—this becomes particularly important since learning processes needed to be sustained irrespective of external circumstances. Considering the complexity of today's health care system, educational resilience should become a strategy. To conclude, hybrid education might prove to be effective in many cases of developing countries.

### 7.4 Future and possible directions

The concept of digital transformation in the education industry in relation to healthcare is still a developing concept. The future will be marked by advances such as artificial intelligence, learning analytics, immersive media, and evidence-based education management.

Artificial intelligence might offer great opportunities to personalize learning paths and adaptive course materials, implement automatic feedback systems, and develop intelligent tutoring applications. Using such technologies will allow educators to discover individual learning requirements and give timely assistance throughout the whole learning period.

The learning/teaching analytics is another one that has potential benefits for clinical education. Learning analytics involves analyzing educational data created within learning management systems and other digital platforms. This can yield important insights into student engagement and educational effectiveness. Immersive technologies such as VR, AR, and enhanced simulations can become more popular for use in clinical training. These technologies can offer safe ways to build students' clinical skills without direct patient interaction, especially in areas where clinical training is scarce.

New types of digital literacy skills are required for future healthcare professionals as well. Apart from technological literacy, it will be important to know about issues such as artificial intelligence, digital ethics, cybersecurity, health data management, telemedicine, and digital healthcare. Medical education organizations should make sure their graduates are ready to work in a digital world of medicine. The experience of YSMU's protests, which shows that digital transformation is not an end goal, but rather a process of continuous adaptation and improvement. It is vital to work together in order for digitalization to contribute to quality and future-proof education of healthcare specialists in the future.

## 8 Conclusion

Digitalization of medical and healthcare education remains a priority in many regions worldwide, but specifically in LMICs. Digitalization of medical and healthcare education remains a priority in many LMICs. The experience of YSMU provides a detailed institutional case of how theory can be translated into practice under real-world constraints. Although the students' attitudes were generally positive, the results cannot be regarded as conclusive proof of improvements in educational quality and clinical competence since they were based on a subjective measure from one institution only. Thus, in 2017, YSMU became one of the first organizations in the Health Sciences and Medical Education Advanced Network (MedNet) to undertake a multifaceted theoretical study of methods and approaches to the digitalization of the health sciences and medical education. The study focused on identifying terminology related to this issue, analyzing the current situation in this field at both the global and regional levels, and developing an action plan for all relevant HSME topic areas. Moreover, the work being done at the institution is further enriched by evaluation results from research conducted across more than 15 regional higher and medical education institutions, focusing on nearly 100 innovative digitalization projects that meet the expectations of all partner universities involved in this network (6, 13)..

## Data Availability

The raw data supporting the conclusions of this article will be made available by the authors, without undue reservation.
